# Prevalence of High Blood Pressure in Pediatric Patients with Sleep-Disordered Breathing, Reversibility after Treatment: The KIDS TRIAL Study Protocol

**DOI:** 10.3390/children9121849

**Published:** 2022-11-28

**Authors:** María Castillo-García, Esther Solano-Pérez, Sofía Romero-Peralta, María Esther Viejo-Ayuso, Laura Silgado-Martínez, Leticia Álvarez-Balado, Rosa Mediano San Andrés, Pilar Resano-Barrio, Francisco García-Rio, Irene Cano-Pumarega, Manuel Sánchez-de-la-Torre, Alfonso Ortigado, Ana López-Dueñas, Laura Fidalgo, Ángel Rodríguez, Olga Mediano, Spanish Sleep Network

**Affiliations:** 1 Sleep Unit, Pneumology Department, Hospital Universitario de Guadalajara, 19002 Guadalajara, Spain; 2 Centro de Investigación Biomédica en Red de Enfermedades Respiratorias (CIBERES), 28029 Madrid, Spain; 3 Pneumology Department, Hospital Universitario La Paz, IdiPAZ, 28046 Madrid, Spain; 4 Sleep Unit, Pneumology Department, Hospital Universitario Ramón y Cajal, Instituto Ramón y Cajal de Investigación Sanitaria (IRYCIS), 28034 Madrid, Spain; 5 Precision Medicine Group in Chronic Diseases, Respiratory Department, Hospital Universitario Arnau de Vilanova y Santa María, 25198 Lleida, Spain; 6 Department of Nursing and Physiotherapy, Faculty of Nursing and Physiotherapy, Universidad de Lleida, IRBLleida, 25002 Lleida, Spain; 7 Paediatric Department, Hospital Universitario de Guadalajara, 19002 Guadalajara, Spain; 8 Medicine Department, Universidad de Alcalá, 28805 Madrid, Spain; 9 Otorhinolaryngology Department, Hospital Universitario de Guadalajara, 19002 Guadalajara, Spain

**Keywords:** sleep-disordered breathing, high blood pressure, sleep apnea, children, cardiovascular risk, adeno-tonsillectomy

## Abstract

Current data support an increase in the prevalence of high blood pressure (HBP) in pediatric patients with sleep-disordered breathing (SDB). Adeno-tonsillectomy has been shown to be an effective treatment for most patients. Our objective was to determine the prevalence of HBP in pediatric patients with SDB and the impact of adeno-tonsillectomy with a multicenter, longitudinal, and prospective study that included 286 children referred for suspected SDB. The diagnosis of SDB was established by polysomnography (PSG) and the diagnosis of HBP by 24-h ambulatory blood pressure monitoring (ABPM). In patients without SDB and SDB without treatment indication, these tests were repeated six months after the baseline visit. For patients with medical treatment for SDB, the tests were repeated six months after the treatment initiation. Finally, in patients with surgery indication, ABPM was performed just before surgical treatment and ABPM and PSG six months after the intervention. The study contributes to elucidating the association between SDB and HBP in pediatric patients. Moreover, it contributes to determining if intervention with adeno-tonsillectomy is associated with BP reduction. The results have direct implications for the management of SDB, providing essential information on treatment indications for existing clinical guidelines. NCT03696654.

## 1. Introduction

### 1.1. Obstructive Sleep Apnea in Children

Obstructive sleep apnea (OSA) is the maximum expression of sleep-disordered breathing (SDB), ranging from simple snoring to OSA [1]. Pediatric OSA is defined as a breathing alteration during sleep, characterized by total (apnea) or partial (hypopnea) obstructions of the upper airway that interfere with normal ventilation and sleep architecture [2]. The severity of OSA is measured by the apnea–hypopnea index (AHI), which shows the number of respiratory events per hour during the sleep. OSA in children is a major public health problem given its high prevalence and its association with relevant consequences, fundamentally in the metabolic, neurocognitive, and cardiovascular spheres [3]. OSA children present nocturnal symptoms such as snoring, nocturnal apneas evidenced by the parents, and enuresis. During the day, the sleep apnea children present with hyperactivity, concentration and memory problems, and somnolence or tiredness [4]. Studies conducted on the prevalence of OSA in children have shown highly variable ratios depending on the population under study, the diagnostic method, and the definitions used. Previous studies have suggested a prevalence of OSA between 1–5% [4,5] in children, with adeno-tonsillar hypertrophy being the most common factor for developing OSA in childhood. A complete polysomnography (PSG) is the diagnostic test for OSA in children that is considered the “gold standard”. Additionally, simplified methods have also been validated in the pediatric population [4].

Although being the same disease, OSA is very different between adults and children: the definitions for respiratory event and OSA severity are distinct, diagnosis tools used in adults have less utility in children, and treatment is usually definitive in resolving the events in children and not in adults.

### 1.2. OSA and Cardiovascular Consequences

During sleep, the patient with OSA is repeatedly subjected to intermittent hypoxia, changes in intrathoracic pressure, and microarousals, which results in the hyperactivity of the sympathetic nervous system, higher oxidative stress, and a proinflammatory and hypercoagulable state [6]. Several studies have suggested that these repeated alterations in each episode of apnea/hypopnea contribute to the development and progression of different cardiovascular diseases, well-documented for high blood pressure (HBP), contributing in these patients to higher mortality and morbidity. In adult patients with OSA, there is an increase in blood pressure (BP) and worse control of it. It has been shown that OSA patients suffer more frequently from a non-dipping pattern and resistant hypertension. The most consistent results relate treatment with continuous positive airway pressure (CPAP) to a reduction in arterial BP [7] and the first signs of atherosclerosis [8]. Randomized clinical trials demonstrate that CPAP treatment reduced blood pressure, was more consistent in resistant hypertension patients and restored the non-dipping pattern [7]. An important aspect is that, to get this effect, a good compliance (better if is more than 6 h per night) is needed [9]. However, the efficacy of CPAP in reducing cardiovascular events has not yet been proven [10,11]. Among the factors that might have influenced this are: (1) the associated cardiovascular illness in OSA adults could be irreversible if treating the disease in adulthood; (2) the presence of cardiovascular risk factors (hypertension, diabetes mellitus, dyslipidemia, etc.) that can act as confounding factors, and (3) the treatment of OSA with CPAP has mainly been suboptimal in this type of clinical trial. For these reasons, the pediatric population constitutes an ideal target group for confirming the impact of OSA on cardiovascular risk prevention: free of established disease and preexisting risk factors, and with effective treatment such as adeno-tonsillectomy. In this way, knowledge of the natural history of the disease would also be facilitated by studying the OSA pediatric population.

### 1.3. Blood Pressure in SDB in Children

Unlike adults, the diagnosis of HBP in children is based on the normal distribution of BP in healthy children and not on the morbidity and mortality associated with it. In this group, ambulatory BP monitoring (ABPM) during 24 h is more favorable than isolated office BP in predicting morbidity and mortality. ABPM offers advantages over isolated measurements since it allows us to study the variability of BP throughout the circadian cycle and it is more appropriate for predicting organ damage. The 2016 European Guideline for the management of high blood pressure in children [12] continues assuming, as reference values, those provided by the US Task Force [13], in which the values are distributed according to percentiles based on gender, age, and height. However, ABPM is complicated to perform in children, given the difficulty associated with this technique obtaining good quality results.

In children with high BP values, even when these are close to normal levels, it is possible to predict the development of hypertension when they become adults [14], cardiometabolic risk [15], and future coronary disease [16].

Alterations in BP in children have also been associated with OSA, although the evidence is limited. Guilleminault et al. [17] were the first to describe higher BP values among children with OSA. However, Zintzaras et al. [18] published a meta-analysis in 2007 in which they concluded that, until that date, there was insufficient evidence about the relationship between SDB and increased BP. Subsequently, ABPM measurements performed in children with SDB reported elevated systolic and diastolic BP, both during the day and night, independent of obesity status [19,20]. To date, few studies have explored how surgery could improve cardiovascular parameters in children with sleep apnea [21,22,23,24,25,26]. A significant decrease was observed in systolic and diastolic BP in children with hypertension and OSA after adeno-tonsillectomy compared to non-hypertensive children [27,28,29,30,31].

Additionally, there is limited evidence related to the physiological mechanism implicated in the cardiovascular risk development in children. The study of specific biomarkers, as is troponin T, has been widely studied in adults [32] and could be a good biomarker for children [33].

From the results provided by these studies, it can be deduced that adeno-tonsillectomy could reduce BP in patients with OSA. However, important limitations in most of them (such as small populations, absence of ABPM in most of them, absence of a control group, etc.) make studies in larger population series and with adequate methodology necessary. For this reason, we set ourselves the main objective of evaluating the BP values present in children with SDB and their response to their treatment.

## 2. Methodology/Design

This article describes the methodology from the registered trial NCT03696654 [34] hypothesis: sleep-disordered breathing increases the prevalence of arterial hypertension in pediatric patients. This hypertension is reversible after treatment.

### 2.1. Primary Objective

To demonstrate how the presence of sleep-disordered breathing (SDB) is associated with a higher risk of high blood pressure (HBP) in pediatric patients and to confirm that this is reversible with treatment.

### 2.2. Secondary Objectives

- Establish the relationship between the presence of HBP and the severity of OSA (apnea–hypopnea index—AHI, desaturation index—DI).
- Evaluate the variability along the circadian rhythm of the HBP patterns produced in pediatric patients with SDB.
- Establish the correlation between the diagnosis of HBP measured in the office and by ambulatory control of BP.
- Assess the organic damage produced:
  - Evaluate the manifestation of subclinical organ damage through other markers such as: blood biomarkers (creatinine/glomerular filtration rate), urine (albuminuria/proteinuria), and echocardiography (left ventricular hypertrophy).
  - Establish the pathophysiological mechanisms involved in the HBP/SDB relationship.

### 2.3. Design and Population

This was a multicenter, longitudinal, prospective study with a control group. A total of 286 children between 4 and 18 years old who were referred prospectively to undergo a sleep study due to suspected SDB were included. The study was directed by the coordinating center (Hospital Universitario de Guadalajara), which was responsible for the study design and patient follow-up. The other participant centers were Hospital Universitario Fundación Jiménez Díaz, Instituto del Sueño, Hospital Universitario Santa Lucía, Hospital San Pedro, and Hospital Universitario de Araba.

#### 2.3.1. Inclusion Criteria

- Approval of the Ethics and Clinical Trials Committee (P02/18).
- Informed consent signed by parents and/or legal guardians.
- Children between 4 and 18 years old evaluated consecutively for suspected SDB.

#### 2.3.2. Exclusion Criteria

- Associated comorbidities: cardiovascular disease (including cardiac malformation), cerebrovascular disease, or unstable severe or exacerbated respiratory disease that preclude the realization of the studies.
- Genetic diseases according to investigator criteria.
- Children with chronic insomnia and/or depressive syndrome.
- Children with malformation syndromes (including craniofacial malformations), Down syndrome, and neuromuscular diseases.
- Previous otorhinolaryngologic surgery and/or CPAP.
- Contraindication for realization of ABPM (arrhythmias, allergy to latex, or coagulation disorders).

Children evaluated for suspected SDB participating in the research study had to meet all the inclusion criteria and none of the exclusion criteria. After informed consent was signed by their parents, the following procedures were developed.

### 2.4. Procedures

Different clinical and anthropometric variables were collected, and the diagnosis of SDB was established by complete PSG and the diagnosis of HBP by taking BP in the office and 24-h ABPM.

#### 2.4.1. Full Polysomnography

PSG was performed according to the criteria of the American Academy of Sleep Studies (AASM 2017). Different signals were recorded, such as nasal flow, snoring, thermistor, thoracic and abdominal movement, transcutaneous capnography, oxygen saturation, heart rate by electrocardiogram, body position, and leg movement. Electroencephalogram recordings will include six electrodes, referred to as contralateral mastoids (A1–A2), adopting the 10–20 rules of international EEG system: two frontal (F3–F4), two central (C3–C4), and two occipital (O1–O2) locations. One ground electrode and another reference electrode (Cz) were included. Two chin electrodes were used to obtain the electromyogram signal and two different electrodes placed above the left and right outer eye cantus were employed to record the electrooculogram (EOG). Apnea is defined as a flow decrease > 90% in two respiratory cycles for obstructive and >20 s or two respiratory cycles accompanied by a desaturation of 3% in central apnea. Hypopnea is defined as a 90–30% flow decrease in two respiratory cycles accompanied by a desaturation greater than 3% or microarousal (AASM 2017). The AHI is defined as the summatory of apneas and hypopneas divided by the sleep time. Based on the results, four groups were created based on the severity of the SDB measured by the AHI: group I: AHI < 3/h; group II: AHI ≥ 3 <5/h; group III: AHI ≥ 5/h <10/h; group IV: AHI ≥ 10/h.

#### 2.4.2. Blood Pressure Measurement

Office BP was measured at the clinic. BP was measured on three occasions, with a pediatric sphygmomanometer validated for pediatric age using the non-dominant arm, the same day as the ABPM (to be used for calibration of the ABPM), and at the post-treatment follow-up visit. The patient needed to be seated for at least 5 min before the BP measures and to remain seated with uncrossed legs and an empty bladder in a quiet environment. Three BP measurements were taken every 3 min, discarding the first one and averaging the last two.

The ABPM study and the BP data collection were done following the recommendations of the European Guide for the management of hypertension in children [12]. Hypertension was considered when systolic blood pressure (SBP) and/or diastolic blood pressure (DBP) were persistently above the 95th percentile according to gender, age, and height and, depending on the distribution of percentiles, the hypertension classification was done. The ABPM was conducted a maximum of fifteen days after the PSG study.

Validated pediatric BP monitoring equipment with sleeves appropriate to the size of the child’s arm was used (the size of the cuff to be used is calculated by the average distance between the acromion and the radial head). The device was placed on the non-dominant arm and parents were instructed on how to handle the device (how to turn it off in case of excessive pressure, need to keep the arm still during measurements) and everything related to the test was explained.

For the study of the circadian rhythm pattern in blood BP, a decrease in SBP and DBP of at least 10% (mean BP during the day − mean BP during the night/mean BP during the day × 100) was considered normal.

### 2.5. Visits and Follow-Up

**Figure 1:**
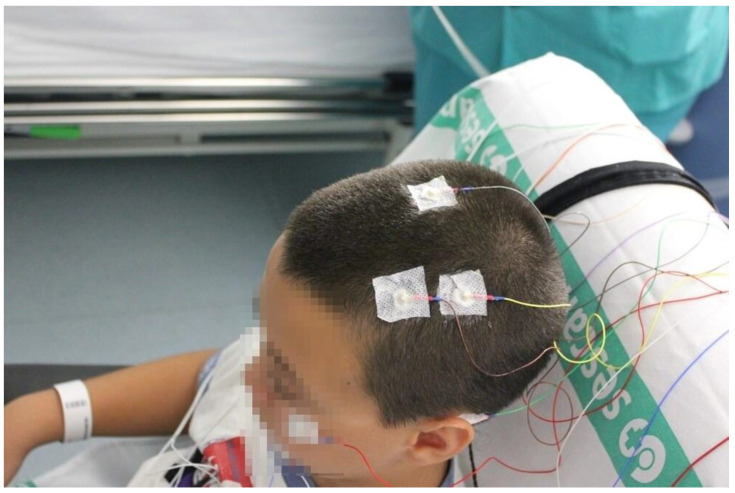
Pediatric polysomnography.

A full sleep study (PSG) was performed at the sleep unit in the basal visit (Figure 1). The night of the sleep study, parents were provided with the Chervin questionnaire, answering questions (Yes/No) related to the child’s behavior both during sleep and while awake. They referred to the habitual behavior of the child in the cardinal or fundamental symptoms of SDB. At the same time, anthropometric measurements were collected by nurses (V1).

**Figure 2:**
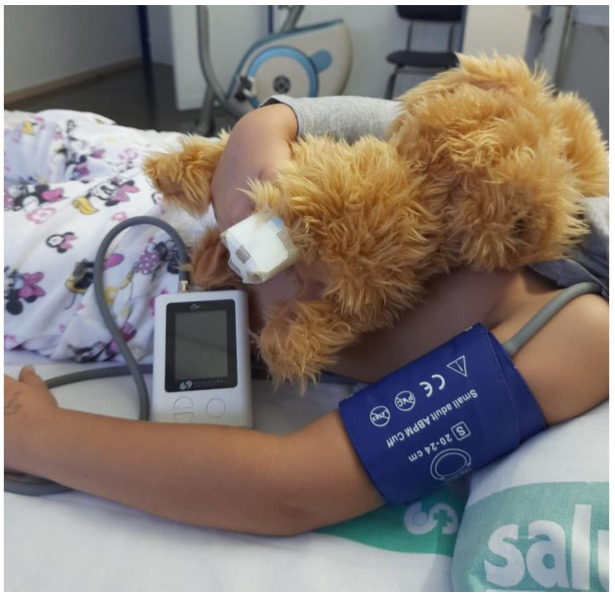
Ambulatory blood pressure monitoring.

For the HBP study, BP was taken in the office and 24 h-ABPM (V2) was caried out in the following hours (Figure 2).

All patients were offered to participate voluntarily in the determination of subclinical organ damage related to hypertension and the pathophysiological mechanisms involved, which were carried out within a maximum period of one month around the performance of each sleep study (and may coincide with the ABPM study).

Once the sleep study was completed, the therapeutic decision was made in the pediatric clinic based on the criteria established in accordance with the SEPAR sleep-disordered breathing in children consensus (V3) [4].

**Figure 3:**
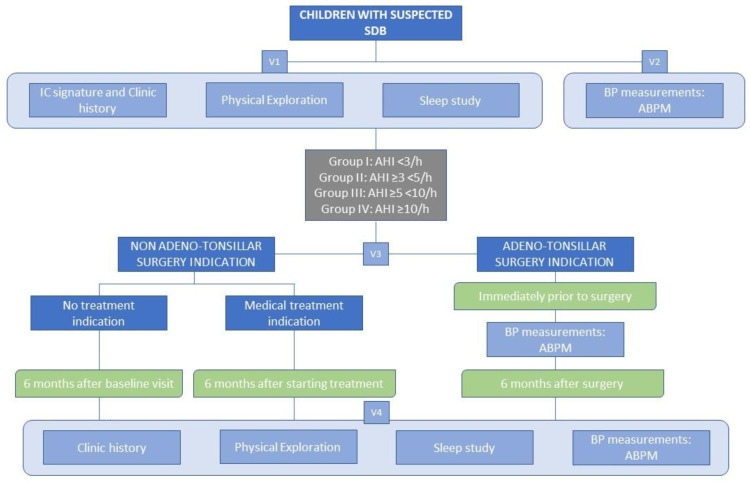
Flow diagram for the study procedure. Abbreviations: SDB, sleep-disordered breathing; IC, informed consent; BP, blood pressure; ABPM, ambulatory blood pressure monitoring; AHI, apnea hypopnea index.

In order to assess the impact of SDB treatment on BP, measurements were repeated after therapeutic application (V4). In patients who do not require treatment or are referred for medical or orthodontic treatment, the tests were repeated 6 months after the therapeutic decision was made (ABPM, PSG, and organ damage studies, if applicable). In patients referred for adeno-tonsillar surgery, the procedures were repeated just before the intervention (ABPM) and six months after it (ABPM, PSG, and organ damage studies if applicable). Thus, a control group was available without impeding the treatment of any patient and without allowing delays in its application linked to the study (Figure 3).

### 2.6. Study Variables and Data Collection

Data collection was carried out in a database created for this purpose. Here, the variables that participate in the study were registered and stored.

The clinical variables were collected through the Chervin questionnaire, a validate questionnaire usually used in research to identify the presence of SDB in children and to identify important symptoms, including related behavioral disturbances, snoring, and daytime sleepiness [35].

In the physical examination, data of weight, height, body mass index (BMI), neck, hip, and waist circumference, micro-retrognathia (Yes/No), Mallampati (I, II, III, III/IV), tonsillar hypertrophy (I, II, III/IV), adenoid hypertrophy (Yes/No), ogival palate (Yes/No), and bite (III/II/open/asymmetric) were taken.

BMI was corrected for weight and height using established guidelines for converting BMI into percentiles. Based on their BMI, children were classified as: underweight (below the 5th percentile); normal weight (between the 5th and 85th percentile); overweight (between the 85th and 95th percentile); or obese (above the 95th percentile). The sleep study variables included were AHI, DI, minimum saturation, percentage of time below 90% saturation (T90), daytime mean saturation, nighttime mean saturation, sleep efficiency, different sleep states (N1, N2, N3-NREM, and REM), arousal number and index, and leg movement number and index. Central AHI (events with absence of thoracic-abdominal movement) and obstructive AHI (events with presence of thoracic-abdominal movement) were included. Polysomnographic variables were collected through PSG performed during the night period. The PSG was valid if it had >300 valid minutes and >180 min of sleep.

The variability of BP in the circadian rhythm was analyzed by means of ABPM for 24 h, collecting data on SBP and DBP in the office, mean SBP (SBPm), and mean DBP (DBPm) globally, during the day and night, and the non-dipper pattern.

Ambulatory BP measurements were taken every 20 min during the day and every 30 min during the night. A study is considered interpretable when it has at least one reading per hour, 50 readings in 24 h, and 65–75% of the programmed readings. The test can be repeated if it is not valid.

For the determination of subclinical organ damage, the following determinations were included: biomarkers (from blood and urine specimens), electrocardiogram, and thoracic echocardiography (left ventricle hypertrophy—LVH data).

The diagnosis of kidney damage due to HBP is principally made by measuring urine albumin and calculating the glomerular filtration rate (calculated according to blood creatinine, age, and height).

LVH is the most extensively documented marker of organ damage caused by HBP in the pediatric group. Early assessment of LVH in children with HBP is currently recommended as it may facilitate primary prevention of cardiovascular disease. The measure used is the left ventricular mass index (LVMI) (g/m^2^), considering LVH when this index is ≥95th percentile (38.6 g/m^2^). Between 30–40% of children with HBP had a LVMI above the 95th percentile and in 10–15% this hypertrophy was severe (>51 g/m^2^).

### 2.7. Sample Size Calculation

The sample size was calculated by taking the values reported by Ng DK et al., as a reference [24]. For an alpha risk of 0.05 and beta risk of 0.2 in a bilateral contrast for repeated measures, assuming the standard deviation of the variable in the reference group and with an estimated loss to follow-up of 20%, 286 patients were required to detect a decrease of 2 mmHg in SBPm.

The analysis was carried out with the programs SPSS version 26.0 (IMB SPSS Statistics, CA, USA) and R 2.6.2 (2008, R Foundation for Statistical Computing, Vienna, Austria), accepting a value of *p* < 0.05 as the significance level. The results were presented as mean ± standard deviation or percentage, according to the type of variable. The adjustment of the quantitative variables to the normal distribution was evaluated using the Kolmogorov–Smirnof test. For comparisons between groups, the chi-square or t-Student tests were used.

### 2.8. Ethical Considerations

This study does not entail relevant risks, except for the discomfort of performing the ABPM study and performing the sleep study control. The ABPM study involves wearing a BP sleeve for 24 h with inflation every 20–30 min. The extraction of biological samples was optional only for those patients who wish to participate in the organ damage sub-study. The study was approved by the Ethics and Clinical Trials Committee (P02/18) and the parents/children signed an informed consent (IC) form. A specific IC was collected for blood and urine samples and biobank storage. Finally, although the study is risk-free, the patients were covered by the general insurance of the National Health System of each participating autonomous community.

## 3. Relevance of the Study

The management of HBP in pediatric patients continues to be a pending issue in routine clinical practice, even though the presence of high BP levels in children (also values close to normal) has been shown to trigger the progression of hypertension in adults and have a significant association with increased cardiometabolic risk and coronary heart disease in the future.

On the other hand, there is evidence of the involvement of SDB in the presence of HBP levels, relating these SDB with the progression of cardiovascular diseases. Adeno-tonsillectomy in children with AOS has been recently described to significantly reduce the BP, similarly to adult CPAP treatment. However, the treatment in adults has not been proven to reduce cardiovascular events.

If it is shown that BP increases because of SDB, and that this is reversible after treatment, this would have direct implications for the management of SDB in children and could provide fundamental information on the treatment indications for existing clinical guidelines. Thus, it is important to assess the correlation between SDB in children and future cardiovascular risk, as it could have a relevant impact in clinical practice.

Besides, this information could have enormous relevance for the management of sleep apnea in adults. If the hypothesis of our studies is confirmed, it would imply that the treatment for OSA in children would have a beneficial effect on reducing their future heart attack risk.

Children, unlike adults, are an optimal population because they have no associated risk factors that could act as confounding factors (naïve condition), they have an effective treatment, and they allow for the natural history of the disease to be studied. As a result, they constitute an ideal target for this research, which will also be useful in the management of adults.

For all the above, we advocate that the KIDS TRIAL study constitutes a clear translational study with high potential clinical applicability value.

## Data Availability

Not applicable.
